# Monaural Sound Localization Based on Structure-Induced Acoustic Resonance

**DOI:** 10.3390/s150203872

**Published:** 2015-02-06

**Authors:** Keonwook Kim, Youngwoong Kim

**Affiliations:** Division of Electronics & Electrical Engineering, Dongguk University-Seoul, Seoul 100-715, Korea; E-Mail: herokim@dongguk.edu

**Keywords:** sound localization, angle of arrival, monaural localization, acoustic resonance, pyramidal horn, cylindrical pipe, fundamental frequency, Cepstrum, discrete Fourier transform, single microphone

## Abstract

A physical structure such as a cylindrical pipe controls the propagated sound spectrum in a predictable way that can be used to localize the sound source. This paper designs a monaural sound localization system based on multiple pyramidal horns around a single microphone. The acoustic resonance within the horn provides a periodicity in the spectral domain known as the fundamental frequency which is inversely proportional to the radial horn length. Once the system accurately estimates the fundamental frequency, the horn length and corresponding angle can be derived by the relationship. The modified Cepstrum algorithm is employed to evaluate the fundamental frequency. In an anechoic chamber, localization experiments over azimuthal configuration show that up to 61% of the proper signal is recognized correctly with 30% misfire. With a speculated detection threshold, the system estimates direction 52% in positive-to-positive and 34% in negative-to-positive decision rate, on average.

## 1. Introduction

The human auditory system offers significantly accurate acoustic imaging compared to a conventional dual-sensor localization system. For the horizontal plane, the localization blur, which represents the smallest change in direction to produce an auditory event, is less than ±10°. Furthermore, a moving sound source in the median plane can be recognized with a localization blur not exceeding ±22° [1]. The extensive use of received information over the magnitude, phase, and frequency provides further accuracy of localization beyond the typical localization system. The notable feature of the human localization function is the spectral cue inserted by the pinna structure. The localization in the median plane is mainly achieved by the perception of the spectral cue over the propagated sound. The symmetric distribution of the aural architecture plays a significant role in the horizontal localization; however, the height variation in median plane cannot be detected without the irregular shape of the pinna.

The pinna reflection was studied by Batteau [2], who explained the shape by direct and indirect sound propagation. The frequency-domain analysis was performed extensively by Shaw and Teranishi [3] using a replica of the external ear as well as natural ears. Afterward, numerous approaches [4–12] have been used to understand the role of the pinna in finding the direction of a sound source. Consistently, the papers have presented the result that the asymmetric structure of the pinna introduces a significant cue based on the spectral modification for direction identification. Upon the received sound, the location of peaks and notches in the frequency information represents the elevation of the sound source.

Understanding the pinna function led to the possibility of a monaural localization (ML) system. A conventional acoustic localizer utilizes the magnitude, phase, and flight time information between multiple sensors; therefore, the system requires at least two sensors for comparison. Generally, the localization performance is proportional to the deployed receiver quantity. The injected spectral modification from the structure around the receiver delivers a fundamental basis for a single-sensor localizer once the system properly estimates the frequency information. Various approaches have been employed for the pursuit of a structure-enhanced localization system as below. Harris *et al.* designed analog circuitry to acquire the time difference between the direct and indirect propagation induced from the pinna structure [13]. The localization dimension of a binaural system was extended to a vertical axis by adapting a pinna-like reflector and cue-detection algorithm [14–18]. Several structures around the microphone were studied for understanding the head-related transfer function in order to enhance a directivity pattern [19]. The bio-inspired pinna structure from a horseshoe bat was investigated for static and dynamic geometrical changes on the acoustic device characteristics. The ML without external structure explicitly exploited the transfer function of indoor speech propagation for environment and speaker dependent localization [20–22]. The ML algorithm was realized by the position-wise transfer function from Cepstral parameters and speech model in the room condition and the performance was further improved by the parabolic reflection structure [23]. A hybrid ML method was suggested by Friedland *et al.* based on the audio-visual approach for unsupervised speaker localization via combining Cepstral parameters with video features [24].

This paper proposes a novel monaural sound localization system based on the spectral cues derived from the unique structure. The basic idea is originated from the nature of the observations investigated and organized by the numerous researchers stated above; however, direct application of the pinna structures has limited performance because of the manner of spectral estimation. The fundamental process of the pinna structure creates direct and indirect acoustic propagation paths in the temporal domain. The finite number of delay components from the pinna shape represent the moving average (MA) model which demonstrates a valley dominant spectrum. The acoustic processing from the pinna is significantly efficient to insert the spectral cues while minimizing the information distortion. On the other hand, the conventional estimation algorithms presents degraded performance compared to brain processing because of incompatibility of the computational model. The limited feasibility arises from the fact that the MA estimation problem is basically a nonlinear one and is significantly more difficult to solve than other model estimation problems [25]. Instead of employing the conventional pinna-resembling figures, the authors utilize an acoustic resonance-inducing shape that provides the multiple peaks in the frequency domain.

**Figure 1.**
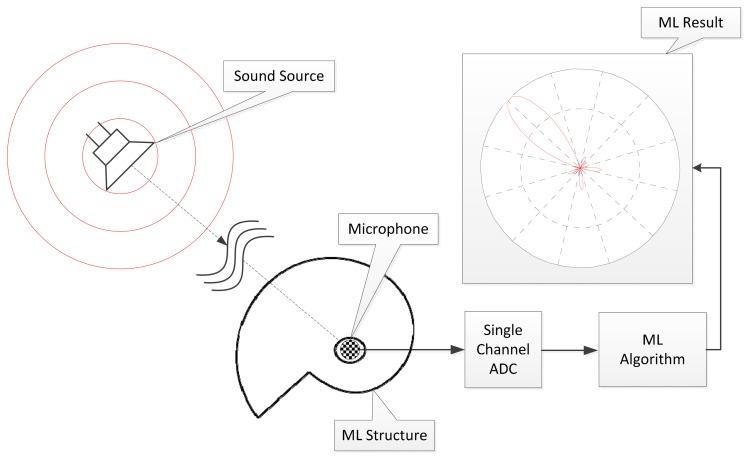
The functional diagram of the ML system.

The acoustic structure used in this paper is pyramidal horns placed around a single pivotal measurement point. According to the angle of arrival (AoA), the acoustic signal travels the different radial lengths of the pyramidal horn that creates the distinctive resonance spectrum known as the fundamental frequency. The localization system utilizes the Cepstrum [26] based algorithm in order to compute the fundamental frequency of the given signal. The major goal of the ML system is to discriminate the spatial direction using spectral cues created by the acoustic passage of the pyramidal horns. The overall functional diagram is illustrated in the Figure 1 with single channel analog-to-digital converter (ADC). The effort of this paper is the extension of the authors' previous works which are the azimuthal movement detection based on binaural architecture [27] and target localization algorithm over the distributed acoustic sensor network [28]. The earlier works concentrated on the acoustic level variations over the sound source movement and this paper appreciates the spectral alternative from the propagation structure. In addition to the acoustic model simulation, the experiments are performed and evaluated within the identical anechoic chamber as the previous works.

## 2. Methodology

The goal of this paper is to design a sound localization system based on a single microphone. A conventional isotropic receiver does not provide any AoA information for localization; hence, two assumptions are required for feasibility. First, the spectrum of the signal must be able to be manipulated in a linear manner before the signal arrives at the receiver. The sound source produces a stationary and broadband signal in order to derive the spectral peaks by the propagation structure in a sufficient time. Second, the indirect propagation according to the diffraction is ignorable. The edges of the structure provide the multipath propagation which causes ambiguity of estimation. However, the algorithm based on the full spectrum dilutes the effect of the frequency-limited diffraction in this work. With established assumptions, the particular structure is applied to the receiver outside in order to control the spectrum of the propagated signal for discrete arrival angles.

Two independent domains must be approached simultaneously to achieve the goal. Those are the optimal structure and the corresponding estimation algorithm. The structure presents the optimal shape which maximizes the distinction between the spectra from a discrete direction. The modified spectrum should be recognizable to the spectral estimation algorithm which represents wide choices in method and model. This paper utilizes the estimation algorithm based on Fourier analysis to understand the structure-derived information. Therefore, the designated asymmetric structure located at the outside microphone and estimation algorithm from the Fourier transform represent the tool chain for ML system. Note that the ML structure indicates the physical structure and the ML algorithm denotes the spectral estimation algorithm with direction model. In addition, the ML system is the combination of structure and algorithm.

The received spectrum from various sound sources exhibits extensive profiles in magnitude and phase. The single frequency modification for direction cue is barely recognized by the estimation process since the algorithm cannot distinguish the difference between the initial and induced spectrum in most of cases. The mutation is performed in a linear manner as a valley or peak over the spectrum magnitude; however, the inserted point is regarded as one of the many generic inflection points in the frequency domain. Unless the system executes post-works such as the temporal processing, which compares the spectra between adjacent time slots based on the stationary signal, the single frequency modification is buried under the source information. This paper leaves the temporal processing for future performance gain and concentrates on the immediate processing; hence, a novel scheme for spectral cue is required for viability.

**Figure 2.**
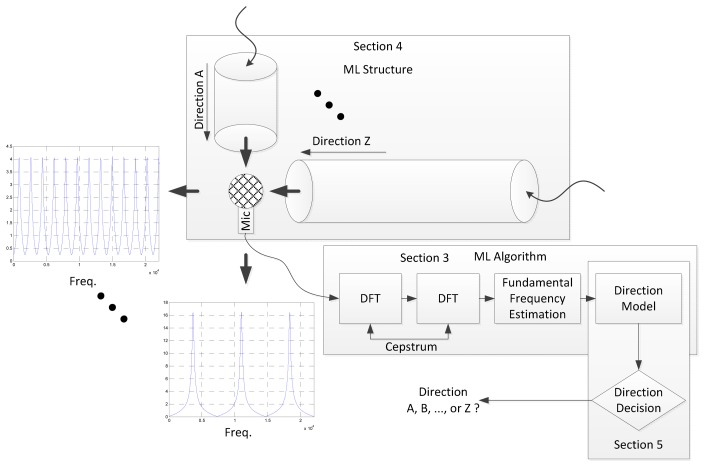
System architecture of proposed ML system with paper organization.

Owing to the structure, multiple frequency peaks are stimulated with even spacing known as the fundamental frequency in this paper. The periodicity in the frequency domain is represented by the placement of peak magnitude in every integer multiple of the fundamental frequency. An asymmetric structure creates the direction-dependent fundamental frequency and the ML algorithm estimates the fundamental frequency and its corresponding direction. The ML algorithm includes the double discrete Fourier transform (DFT) for fundamental frequency and direction model for propagation length. The direction model is the mathematical model between fundamental frequency and propagation length. The length is simply evaluated by the direction decision for final AoA from structure profile. The overall diagram of the ML system is illustrated in Figure 2 and further detail of the procedures can be found elsewhere in the paper, as follows: the Cepstrum-based ML algorithm is provided and validated for a conventional cylindrical pipe in Section 3. The ML algorithm is verified with acoustic experiments for estimation performance that offer design parameters for the ML structure. Section 4 designs the ML structure to generate a discrete fundamental frequency for individual azimuthal directions. The approximated model and analysis are demonstrated as well. Section 5 realizes the physical ML structure for the acoustic experiment and analyzes the consolidated ML system for overall estimation. The performance is described by estimating the error and producing a receiver operating characteristic (ROC) curve.

## 3. ML Algorithm Design

This section provides the mathematical basis for estimating the fundamental frequency by using the Cepstrum algorithm. The algorithm is verified via implementing the experiment with a cylindrical pipe which delivers a well-defined acoustic resonance. The given relationship between the fundamental frequency and pipe length is utilized to assess the tube dimension from the recorded signal in the anechoic chamber. The experiment results serve fundamental basis to determine the design parameters of the dedicated structure for ML system in Section 5.

### 3.1. Cepstrum-Based Algorithm

An estimation of the fundamental frequency is initiated from the periodicity in the frequency domain. The Cepstrum is introduced and utilized to estimate the cycle frequency between the peak responses in the spectral domain. The following equations demonstrate the Cepstrum for the fundamental frequency estimation procedure:

(1) $$X[k]=\sum_{n=0}^{N-1}x[n]e^{-j\frac{2\pi }{N}kn}$$

(2) $$\hat{X}[r]=\sum_{k=0}^{\frac{N}{2}-1}\log_{2}\left|X[k]\right|e^{-j\frac{2\pi }{\left(\frac{N}{2}\right)}rk}$$

(3) $$r_{\text{max}}=\underset{r=\left\{r\in \mathbb{Z}|0<r\leq \frac{N}{4}\right\}}{\text{arg}\;\text{max}}\left|\hat{X}[r]\right|$$

For a given time sequence *x*[*n*], Equation (1) denotes the DFT with data length *N*. In Equation (2), the second transform performs the DFT over the logarithm magnitude of the frequency information. The outcome of the second transform contains the fundamental frequency found by Equation (3). A higher *X̂*[*r*], magnitude represents a stronger periodicity in the frequency distribution of *r* value; hence, by applying the maximum argument operation, the fundamental frequency is estimated in Equation (3). The transform given by Equations (1) and (2) performs the operations based on an integer number for the input and output sequence. A proper interpretation is required to obtain the physical unit from the index and is expressed as:

(4) $${\tilde{f}}_{\text{fund}}=\frac{f_{s}}{2r_{\text{max}}}$$

where *f_s_* is the sampling frequency of the given *x*[*n*] sequence and *f̃_fund_* is the estimated fundamental frequency. A detailed derivation of Equation (4) is shown in Appendix A.

### 3.2. Cylindrical Pipe Model

By acoustic resonance, the frequency response of the pipe is maximized for every integer multiple of the frequency that corresponds to the twofold size in the wavelength greater than the effective pipe length. While one end of the pipe is excited by the acoustic system speaker, the mechanical impedance at the boundary is the radiation impedance of the open end, which is non-zero. By adopting an end-correction factor, the equation for the fundamental frequency of the open-ended cylindrical pipe is illustrated as [29]:

(5) $$f_{\text{fund}}\left(L\right)=\frac{c}{2\left(L+\frac{4d}{3\pi }\right)}$$

where *c* is the sound speed, *L* is the pipe length, and *d* is the pipe diameter. Once the fundamental frequency is acquired, the length of the pipe is determined by the preceding equation. The estimated length *L̃* is computed by the following equation, which is derived from Equations (4) and (5):

(6) $$\tilde{L}=\frac{c}{f_{s}}r_{\max}-\frac{4d}{3\pi }$$

### 3.3. Experiment with Cylindrical Pipe

**Figure 3.**
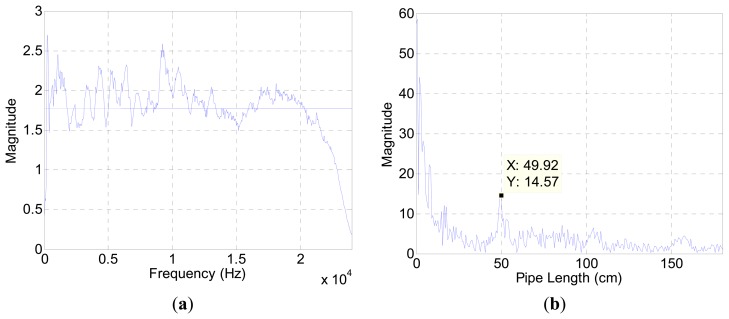
(**a**) Spectrum plot without a pipe based on the white noise; (**b**) Length estimation plot with a 50 cm pipe.

The acoustic experiment was performed in an anechoic chamber with measurement equipment described in Appendix B. The recorded sound includes the pipe attributes as well as the speaker, microphone, and chamber properties. Figure 3a displays the spectrum plot without the pipe structure based on the generated white noise. The white noise is fabricated from normally-distributed pseudorandom numbers in the Matlab software, and the term “white” is only preserved within the discrete data in the computer.

**Figure 4.**
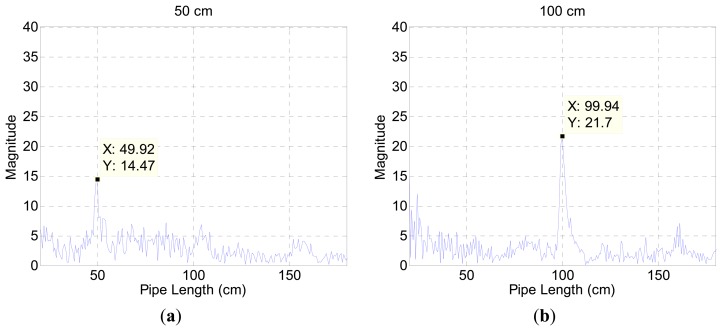
Range-limited output plot (**a**) for a 50 cm pipe; and (**b**) for a 100 cm pipe.

The fluctuation away from the average line in Figure 3a represents the contributions of audio devices and evidence of spectral colorization. Figure 3b illustrates the estimation result for a 50 cm pipe in the chamber. The outcomes below the 20 cm length expose the high magnitude which impairs the estimation process. A short length pipe provides a high fundamental frequency according to Equation (5). The periodicity of the relatively wide frequency stride in Figure 3a participates to generate short-length pipe values in Figure 3b. Since the high fundamental frequency is an intrinsic property of the measurement environment, the algorithm is constrained to estimate a 20 cm pipe and above in length. Figure 4 presents range-limited output plots for the two pipe-length configurations.

For further experiments, the target pipes are of 16 lengths, from 25 cm to 100 cm, each increasing in length in 5 cm steps, with a fixed 3.6 cm diameter. The actual length of the pipe is measured with a laser distance meter (DISTO D3a, Leica, Heerbrugg, Switzerland) with 1 mm precision. The sound is gathered for approximately 20 s with a sampling rate of 48 kHz at an ambient temperature of 19.5 °C, and a corresponding sound speed of 343 m/s. The Cepstrum length is 1024 samples, and ten iterations are ensemble averaged to reduce the variance of the transformation. The individually recorded sounds provide the 87 results, which establish the deterministic outcomes due to the stationary experimental condition. The collective results are organized in Table 1, in which the first row *L* is the measurement, the second row *L̃* is the estimation, and the third row |*e*| is the discrepancy between the two as an absolute value.

The mean estimation error from the results is 0.6 cm, with a 0.3 cm^2^ statistical variance. Relatively short length pipes have less accurate performances. Short length pipes possess a high fundamental frequency, which is easily contaminated by the acoustic measurement conditions.

## 4. ML Structure Design

**Figure 5.**
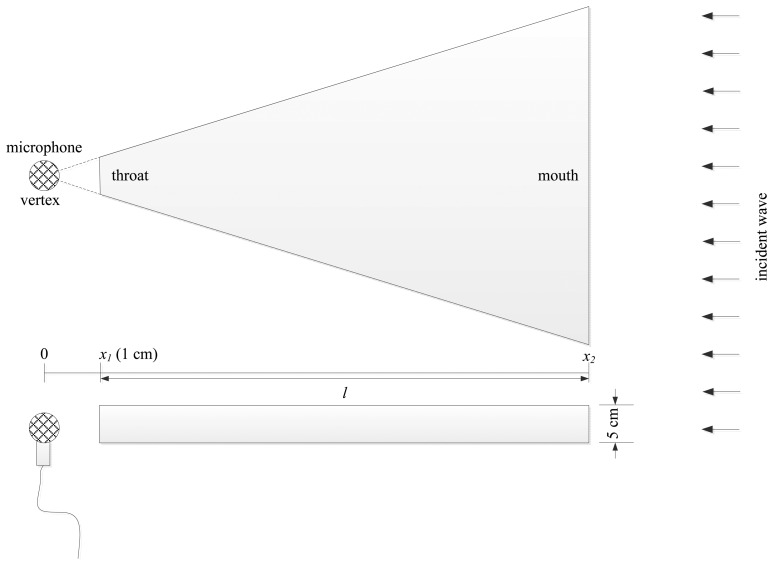
Individual pyramidal horn design.

A cylindrical pipe is well suited for generating a specific fundamental frequency by modifying the longitudinal length; however, multiple pipes cannot be placed around the single receiver because of limitations in the physical dimensions. For complete azimuthal monitoring, a shallow cylindrical shape is radially divided into a number of estimation resolutions and each section is characterized by a pyramidal horn shape as shown in Figure 5. The pyramidal horn is expected to exhibit the acoustic resonance for a lengthwise fundamental frequency that can be estimated by the ML algorithm. The mathematical relationship between the fundamental frequency and the radial length provides a closed-form equation as direction model for outcome translation.

This section investigates the characteristics of the individual pyramidal horn structure from the view point of fundamental frequency. The term “individual” is used since the final structure with multiple horns may cause a correlation effect which is explored in the next section with acoustic experiments. The mathematical complexity of the overall ML structure is significantly high and beyond the analysis of this paper. The single pyramidal horn is analyzed in terms of the pressure amplification factor for the range of frequencies. The sound source is located far from the horn mouth side; hence, the incident wave toward the structure is assumed to be a plane wave signal. The receiver is placed 1 cm away from the horn throat as shown in Figure 5. Note that the radial length of the pyramidal horn is the shortest distance between the throat and mouth, shown *l* in the figure.

The mathematical model for the pyramidal horn with a given configuration is not explored extensively in the literature as of the writing this paper. We note that a derivation of the model is not the object of this paper; hence, the conventional conical horn model is instead adapted for the structure analysis. Recently, Donskoy and Cray developed a mathematical model for receiving acoustic horns including a conical horn in order to achieve velocity amplification [30]. The paper presented the following equation for a sound pressure amplification factor that is the ratio between the sound pressure at throat *P*_1_ and unperturbed medium *P*_0_. To utilize the given equation, the cross section area of throat and mouth is equalized for both shapes and other parameters are directly applied without modification. A further detailed description of the equations and parameters are given in Appendix C:

(7) $$\frac{P_{1}}{P_{0}}=\rho_{0}c_{0}\left(\frac{1-\left(a-cZ_{02}\right)e^{-\text{jkl}}}{a+\frac{b}{Z_{01}}-cZ_{02}-d\frac{Z_{02}}{Z_{01}}}\right)$$

**Figure 6.**
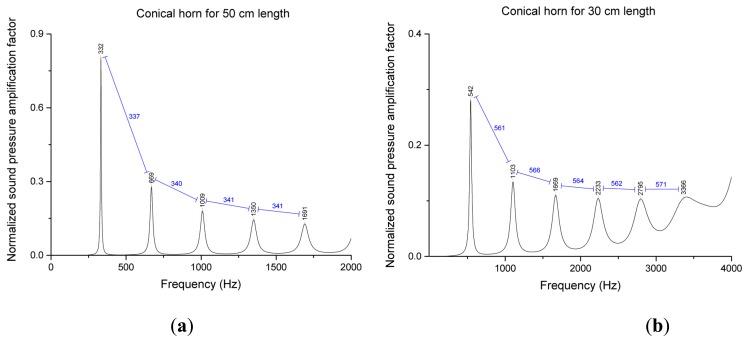
Normalized sound pressure amplification factor for conical horn with (**a**) 50 cm radial length; and (**b**) 30 cm radial length.

The normalized sound pressure amplification factor from the preceding equation is plotted for the 30 cm and 50 cm conical horns in Figure 6, which denotes a limited range of frequency. Both figures represent a fundamental frequency component in the spectral domain. At approximately 340 Hz and 560 Hz the fundamental frequency is observed in the plot for the 50 cm and 30 cm conical horn, respectively. The inverse proportional relationship is noted as similar to Equation (5) for cylindrical pipe. The ML algorithm estimates the actual spectral distance between peaks and the derived proper model translates into the corresponding length and direction. The mathematical model for fundamental frequency and radial length of the pyramidal horn will be developed and analyzed within the complete ML structure in the next section.

**Figure 7.**
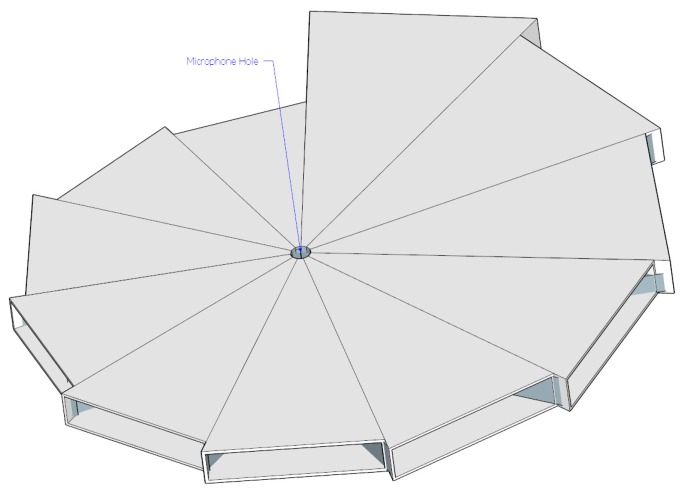
Designed ML structure.

In Table 1, the ML algorithm experiment with cylindrical pipes shows a maximum 1.4 cm estimation error for 30 cm and longer pipes; hence, the design of the ML structure uses a 2 cm radial resolution for pyramidal horns. The entire azimuthal plane is divided into 11 directions which consist of pyramidal horns increasing from 30 cm up to 50 cm gradually. The estimation angle resolution from the given structure is 32.7° and the assembled structure is shown in Figure 7. The hole in the structure body is a 2 cm diameter circle reserved for the acoustic receiver. The individual arrival angle determines the propagation length to the receiver and the distinctive fundamental frequency that is expected.

Cardboard with 0.5 mm thickness is used in the construction of the designed ML structure. The accuracy is developed by a large scale printer and CAD program used to print the sketch of the individual pyramidal horn over the cardboard. The entire ML structure is completed by the 11 horns assembled together as shown in Figure 7. The simple architecture and oversized scale of the ML structure choose the origami style realization instead of 3D printing technology. Note that the previous paper [27] achieved a medium-size binaural localization structure via a filament based 3D printer. The ML structure is placed by the holding device for further experiments, described in the next section.

## 5. Results

**Figure 8.**
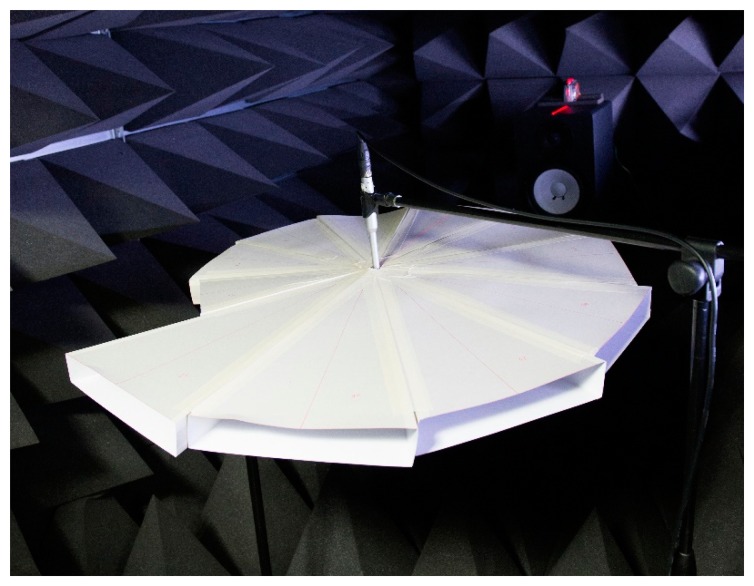
Acoustic experiment with ML structure.

Prior to executing the performance analysis on the designed ML system, further experimentation is required in order to determine the relationship between the fundamental frequency and ML structure direction. The previous section presents the mathematical model for the discrete conical horn by Donskoy and Cray [30] and verifies the existence of the fundamental frequency by showing the spectral plots. Above the linear combination of the horns, the ML structure shows an additional relationship that is investigated in this section. Therefore, the first part of this section is devoted to the acoustic experiment on the ML structure direction and corresponding fundamental frequency for direction model. As shown in Figure 8 and Appendix B, the distance between the microphone end and speaker drive is 1.2 m to maintain the acoustic far-field provision. Also, the direction precision is ensured by a vertical laser surface from the level meter on the speaker body. The acoustic signal for each ML direction in the pyramidal horn is assumed to be transmitted in a radial pathway through the middle of the horn.

**Figure 9.**
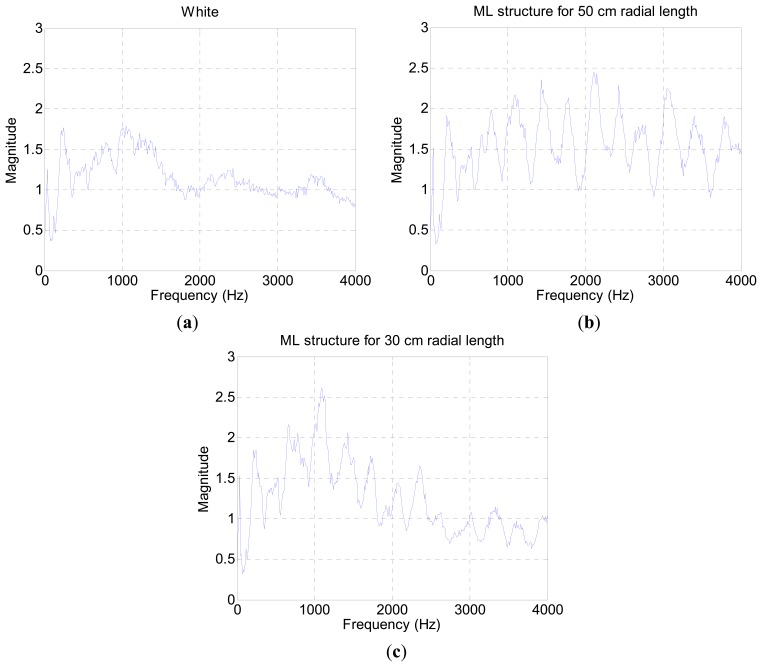
Frequency response of (**a**) anechoic chamber; (**b**) ML structure for 50 cm length; and (**c**) ML structure for 30 cm length.

Besides the empty chamber response, all 11 acoustic data are collected for ML direction model from 30 cm up to 50 cm pyramidal horns every 2 cm apart. Figure 9 illustrates the shortest section, longest section, and empty chamber response based on the white noise signal. The empty chamber response, Figure 9a, shows a relatively flat magnitude for the given frequency range. The coarse fluctuation is expected to provide a high fundamental frequency in the ML algorithm. In order to avoid mutual interference, the ML structure generates a fundamental frequency that should be low enough for proper estimation. Compared to the chamber response, the ML structure results explicitly exhibit the periodicity in the frequency domain from a regular peak and valley distribution. Figure 9b,c demonstrate two extreme paths of ML structure and the inversely proportional relationship is observed in fundamental frequency by measuring the peak to peak distance.

**Figure 10.**
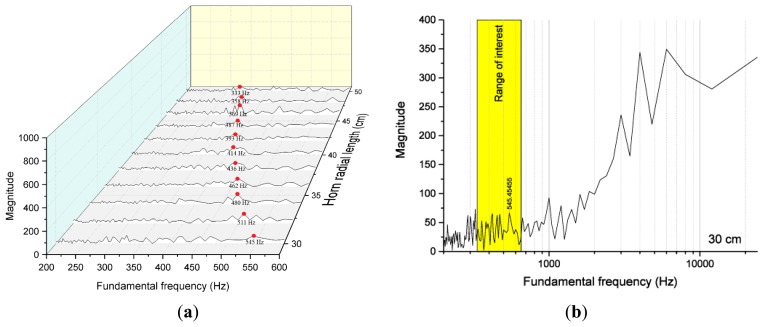
(**a**) Range-limited ML algorithm output for each radial length; and (**b**) entire output for the 30 cm radial length with a yellow shaded range for interest.

From the given data set, the ML algorithm generates outcomes for the series of fundamental frequencies; however, not all results are valid for evaluation. Figure 10a,b show the 3D plot of 11 outputs for a limited range as well as the 30 cm path consequence for the entire fundamental frequency, correspondingly. The full range plot represents a more significant magnitude in the high fundamental frequencies than the value around several hundred that is the expected fundamental frequency for the 30 cm horn shown in Figure 6b. The conventional ambient environment causes the relatively smooth and wide frequency variation depicted in Figure 9a and the spectral property contributes to the generation of increased values in the upper fundamental frequency area. The structure-induced fundamental frequency is comparatively low in accordance with Equation (5); therefore, the seeking range is limited for ML structure as represented by the yellow area in Figure 10b. Note that the derived maximum magnitude for the fundamental frequency is indicated by a red circle in Figure 10a.

Equation (5) provides the mathematical model between the fundamental frequency and cylindrical pipe [29] and Donskoy & Cray calculate the model for a conical horn [30]. Both equations cannot be applied directly to the ML structure because of the discordance of shape parameters. The pyramidal horn in the ML structure has two open ends and one radial length related to the fundamental frequency computation. The throat size is fixed to be constant but the mouth size including the radial length are both unknown within the equation; hence, instead of utilizing the established model, a length-wise first-order rational equation is employed and served as direction model in Equation (8), where *c* is the speed of sound and *l* is the propagated radial length of the ML structure. The constant coefficients for the numerator and denominator are evaluated by the least-square method described in Appendix D:

(8) $$f_{\text{fund}}=\frac{1.0482c}{2\left(l+3.4968\right)}$$

**Figure 11.**
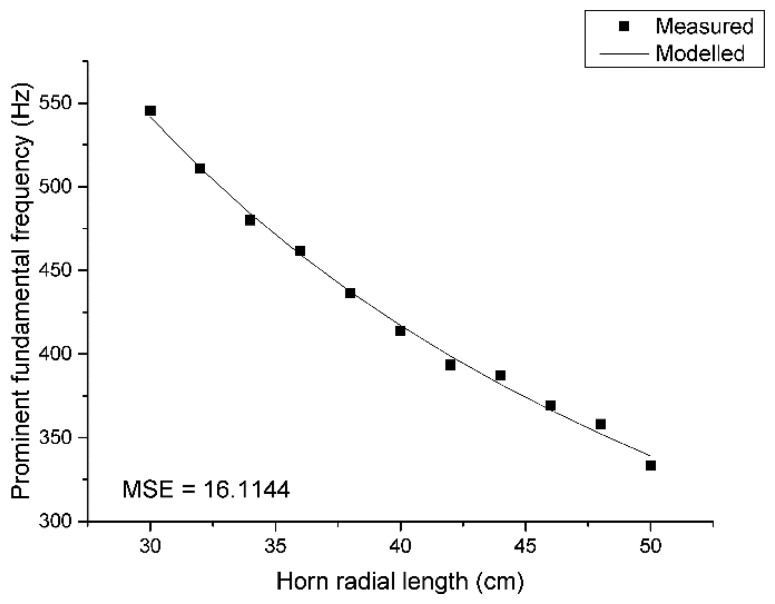
The measured and modelled fundamental frequency for ML structure lengths.

Figure 11 describes the accuracy of the model by placing the measured and modelled data together. The mean square error is 16.1144 Hz^2^ and the modelled line follows well the measured points in the figure. Once the fundamental frequency is obtained by the ML algorithm from the acquired data, Equation (8) computes the pyramidal horn length corresponding to the ML direction. Note that the measured fundamental frequencies are developed from the ML structure which is the collection of 11 individual pyramidal horns, as shown in Figure 7. Each horn may produce the deviated fundamental frequency from the model and discretion is required for use of the modified structure.

**Figure 12.**
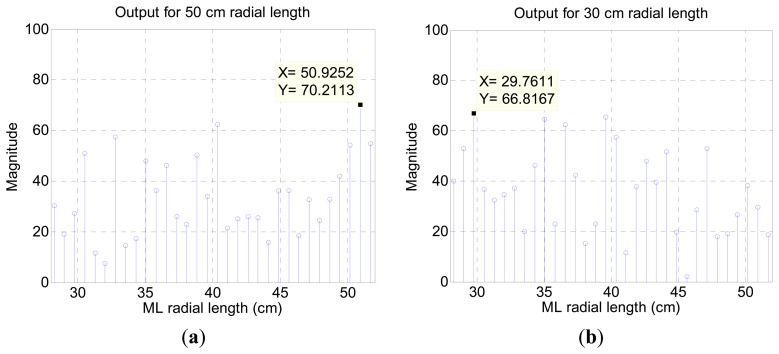
ML algorithm output (**a**) for 50 cm radial length; and (**b**) for 30 cm radial length of ML structure.

From the 30 cm and 50 cm incoming signals, Figure 12 visualizes the likelihood of the propagation length by utilizing Equation (8) for the ML algorithm. The increased value presents higher probability of travel length; therefore, the maximum value in the figure signifies the arrival radial length and direction. The length of the horn is discrete every 2 cm but the output of the algorithm provides finer grain discontinuity because of the ML algorithm resolution and Equation (8). The range of threshold is used to match the nearest actual length in the decision process.

**Figure 13.**
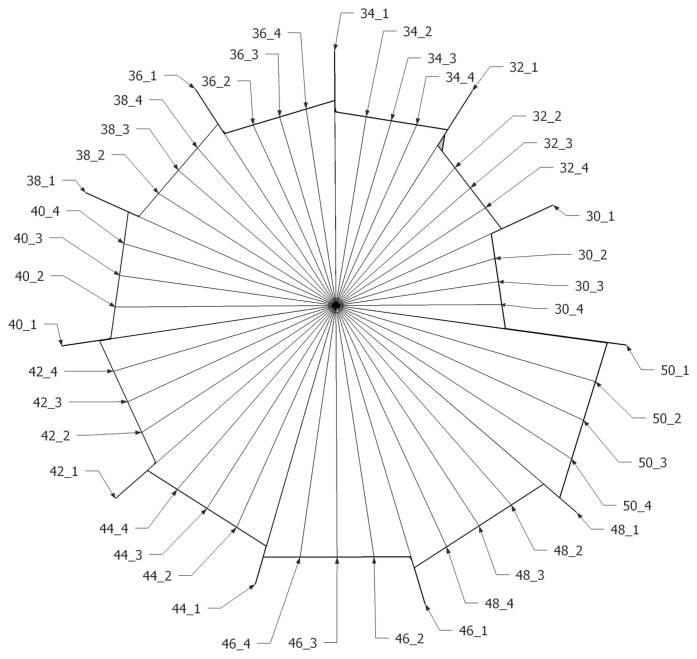
Direction index. ##_% denotes that ## is radial length and % is sub-index.

Compared to the cylindrical pipe results shown in Figure 4, the ML outcome in Figure 12 reveals a relatively inconspicuous distribution that escalates the possibility of false decision. For the identical radial length 50 cm, the ML result presents moderate signal to noise ratio (SNR) because of the varying sectional area from pyramidal horn. The distribution is primarily determined by the resonator shape [31] and the fundamental frequency is controlled by the structure length. The author chooses the pyramidal horn for monitoring the entire azimuthal direction without a complicated and bulky architecture. Based on the algorithm and model, the ML structure is radially divided into finer directions for further experiments, as shown in Figure 13. The index for each direction is identified by showing the radial length first followed by the sub-index number. The sub-index 1 indicates the path with a neighboring horn and 3 specifies the exact center of the given pyramidal horn. With 11 horns around the microphone, the directions for experiments are increased to 44 as below. The sub-index structure lines in Figure 13 are virtual to indicate corresponding directions. Note that the previous pilot experiment was performed in the directions of ##_3 only and the result precisely agreed with the predicted fundamental frequency and horn length. The subsequent study with finer directions helps to understand the influence of the off-center propagations.

Table 2 organizes the result of the ML system experiments for 44 directions. Since both ends of the horn have a linear shape, the off-center propagations travel a farther distance than the center line shown as ##_3. The third column of the table reflects the actual geometry and provides a real physical distance. Also, ##_1 denotes the transition section between two discrete horn lengths; therefore, the actual length is not defined (ND) in the column. The estimated length is calculated from the proposed ML algorithm and the error in the last column is the absolute difference between the actual and estimated distance.

The centers of the horns (##_3) are used to calculate the mean 0.24 cm and variance 0.03 cm^2^ in error. The off-center values yield a mean 3.27 cm and variance 18.95 cm^2^ in error. The data from the centers are referred to the model construction; hence, the results deliver further accuracy, as anticipated. The deviated propagations such as ##_2 and ##_4 produce high and extensive estimation errors since the wide structure is more susceptible to a vulnerability of fundamental frequency contamination. Statistically, 59% (13/22) of the off-center values exhibit less than 1 cm estimation error, which is the range within the proper direction estimation. The deterioration is originated from the 18% (4/22) significant errors which are more than 10 cm estimation error. Although the error is not defined in the transition ##_1, certain results exhibit the proper estimation that is the middle length between the two horns.

**Figure 14.**
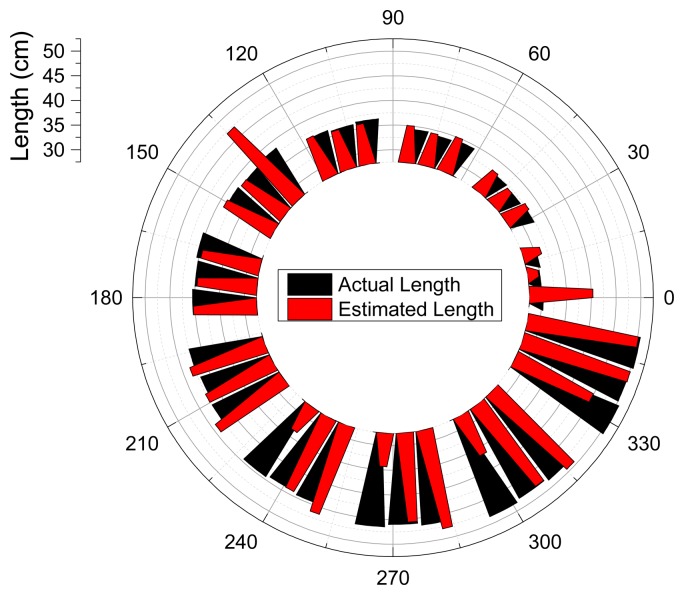
ML system performance plot.

To understand the spatial correlation, Figure 14 visualizes the actual and estimated length for corresponding directions. Black and red triangles are stretched toward the radial path up to its actual and estimated length, respectively. Note that the transition directions ##_1 are excluded and only center and off-center values are illustrated in the depiction. No significant spatial pattern is observed in the figure and the substantial discrepancies in length are randomly located in all directions. The short (high), middle (central), and long (low fundamental frequency) horns have the prominent errors. Another upshot of the estimation is the appearance of comparable performance below with dislocation of the pronounced error directions.

**Figure 15.**
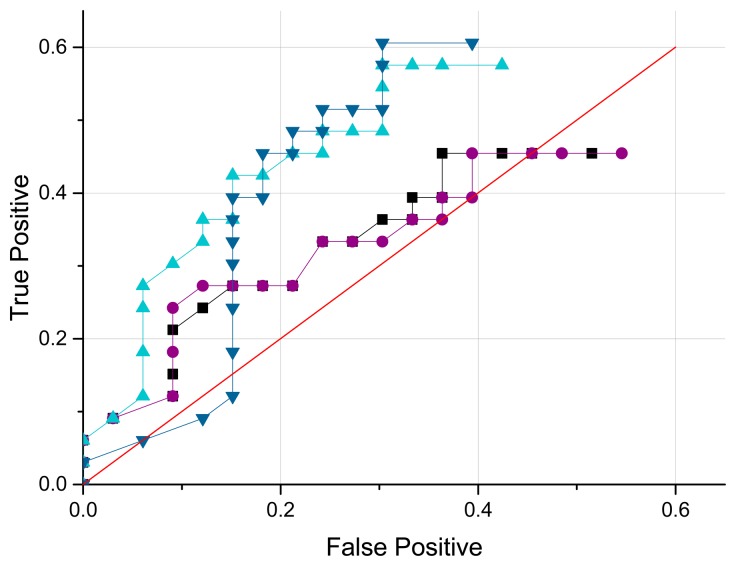
ROC curves for ML system.

Out of 30 experiment sets, the randomly selected data are processed for ROC in Figure 15. Because of the multiple adjacent horns in the ML structure, the number and range of decision thresholds are distinct from the conventional ROC operations which have a single and unlimited range of decision thresholds. The true positive rate (TPR) is the ratio of the number of true positives to the number of positive conditions. The false positive rate (FPR) is derived from dividing the number of false positives by the number of negative condition. One experiment data set includes the entire 33-direction ensemble of acoustic signals without the transition directions. A positive condition indicates that the acoustic signal properly determined the intrinsic direction within the acceptable error range that is 1 cm in absolute value. However, a negative condition specifies the incoming signal above the allowable error range. As the decision threshold is changed from minimum to maximum, one portion of the signal is arranged into a positive outcome and another portion is classified into a negative outcome. The conventional ROC curve drives the decision threshold up to the maximum that is the value yielding both TPR and FPR of one. Because of the adjacent horns, the ML ROC pushes the threshold up to 1 cm which means the decision range is below and above one centimeter away from the actual length of the horn. For example, a 40 cm horn can have the 39 cm and 41 cm decision level as maximum. Note that beyond the range there is an overlap and ambiguity between neighboring horns due to the 2 cm design gap.

At a decision threshold 0.84 cm, the highest performance can be seen as the dark blue triangle located on top left side; TPR is 0.61 and FPR is 0.30. In the identical level, the other curves show 0.58/0.30, 0.45/0.37, and 0.45/0.39 in TPR/FPR form. All of the curves produce the best performance in terms of highest TPR and lowest FPR in the threshold 0.84 cm. The average of TPR/FPR is 0.52/0.34. The threshold value can be increased up to 1 cm with a cost of increasing FPR. Therefore, the overall results suggest that the actual length ±0.84 cm is the suitable bound to estimate direction based on the ML system.

## 6. Conclusions

This paper presents a novel method to localize the arrival angle of sound with a single microphone. An acoustic resonance architecture is used to discriminate between angles. A pyramidal horn structure provides a length-wise fundamental frequency in an inversely proportional manner; therefore, 11 horns with discrete radial lengths are located around the receiver in order to provide angle-wise fundamental frequency. The combination of horns exhibits an asymmetrical circle shape in order to determine source angle in the azimuthal plane. The Cepstrum-based algorithm estimates the fundamental frequency of the incoming signal and the mathematical model between the fundamental frequency and horn length derives the corresponding length and angle. The results show that up to 61% of the proper signals are recognized correctly with 30% misfire. With a speculated error threshold of 0.84 cm, the ML system estimates direction 52% in positive-to-positive and 34% in negative-to-positive decisions rate on average.

Using both a pyramidal horn and a Cepstrum-based algorithm, the ML system demonstrates a relatively valuable performance in obtaining proper direction. Conventional localization systems utilize multiple sensors with tightly bounded architectures and highly computational algorithms. Therefore, the complexity of the system is increased exponentially in terms of system parameters. The ML system not only provides adequate performance but also requires low complexity in computation and hardware. The additional structure is placed around the receiver in order to produce the anisotropic properties that are recognized by the algorithm.

The particular structure and algorithm improve SNR of the ML result for high estimation probability. The choices of acoustic structure and estimation algorithm are numerous. In addition to the continuous structure for high resolution localization, the future works will involve the adoption of modified tubes such as spiral, bended, and vented pipes. Furthermore, the sphere structure for Helmholtz resonance will present the suitable choice for modifying the incident wave spectrum. This paper uses a non-parametric spectral estimation algorithm to process the signal based on the Fourier transform. The future works will include a parametric method in order to compute the frequency information by using a mathematical model and its coefficients. The temporal post-processing of spectral information will explore the similarity between consecutive data set to increase the statistical performance. Lastly but shortly, reducing the structure size will be essential topic in next paper. With all above, the 3D ML for azimuth and elevation direction is the final objective of this research.

The proposed concept of this paper is originated from the human auditory system that localizes the altitude of sound source using the pinna structure. The principal idea is appreciated but realization of the method in a ML system is fabricated based on the understanding of acoustic propagation and spectral estimation. Typical bio-inspired localization systems attempt to mimic the structure and algorithm in the way the nature does. The novelty of this paper is not limited to proposing a method well suited for a general engineering architecture with high feasibility. Also, as 3D printer technology accelerates, we may broaden the structure design span that creates the desired acoustic signal by simple computer aided software. The authors believe that this ML paper contributes to the growth of a certain branch of acoustic localization and hopefully expands the population of publication by more researchers in the future.

## Figures and Tables

**Table 1.** Experiment results in centimeters.

*L*	25.1	30.1	35.1	40.1	45.1	50.0	55.0	60.1

*L̃*	27.1	31.3	36.3	39.9	44.9	50.6	55.6	60.6
|*e*|	2.0	1.2	1.2	0.2	0.2	0.6	0.6	0.5

*L*	65.0	70.0	75.0	80.0	85.0	90.1	95.0	100.0

*L̃*	66.4	69.9	74.9	79.9	85.7	89.9	94.9	100.7
|*e*|	1.4	0.1	0.1	0.1	0.7	0.2	0.1	0.7

**Table 2.** ML system performance results. (ND is Not Defined.)

**Nominal Length**	**Sub-Index**	**Actual Length**	**Est. Length**	**Error**
30	1	ND	32.78	ND
	2	30.32	31.27	0.95
	3	30.00	29.76	0.24
	4	30.32	40.34	10.02

32	1	ND	34.30	ND
	2	32.34	32.78	0.44
	3	32.00	32.03	0.03
	4	32.34	32.78	0.44

34	1	ND	36.56	ND
	2	34.56	35.05	0.69
	3	34.00	34.30	0.30
	4	34.56	35.05	0.69

36	1	ND	39.59	ND
	2	36.38	36.56	0.18
	3	36.00	35.81	0.19
	4	36.38	35.81	0.57

38	1	ND	38.83	ND
	2	38.40	38.83	0.43
	3	38.00	38.08	0.08
	4	38.40	47.15	8.75

40	1	ND	29.76	ND
	2	40.42	40.34	0.08
	3	40.00	39.59	0.41
	4	40.42	39.59	0.83

42	1	ND	45.63	ND
	2	42.44	44.12	1.68
	3	42.00	42.61	0.61
	4	42.44	43.37	0.93

44	1	ND	34.30	ND
	2	44.47	46.39	1.92
	3	44.00	44.12	0.12
	4	44.47	33.54	10.93

46	1	ND	44.88	ND
	2	46.49	47.90	1.41
	3	46.00	45.63	0.37
	4	46.49	34.30	12.19

48	1	ND	47.90	ND
	2	48.51	49.41	0.90
	3	48.00	47.90	0.10
	4	48.51	36.56	11.95

50	1	ND	47.90	ND
	2	50.53	50.17	0.36
	3	50.00	50.17	0.17
	4	50.53	44.88	5.65

**Figure A1.**
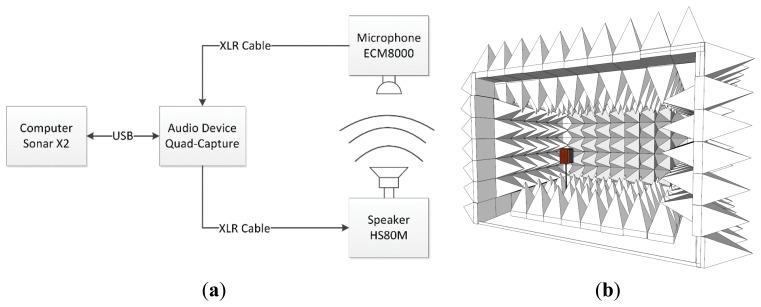
(**a**) Measurement configuration; (**b**) Perspective sketch of anechoic chamber.

## Appendix A.

### Axis Representation of the Cepstrum-Based Algorithm

The spectral resolution from the first DFT is given by Δ*f*, and the axis scale of the first DFT is derived as:

(A1) $$\Delta f=\frac{\frac{1}{T_{s}}}{N}=\frac{f_{s}}{N}$$

(A2) $$\text{axi}s_{1\text{st}\;\text{DFT}}\left(k\right)=\text{k}\Delta f;k=\left\{k\in \mathbb{Z}|0\leq k\leq \frac{N}{2}\right\}$$

where *T_s_* is the sampling period, *f_s_* is sampling frequency, and *N* is the first DFT length. The resolution from the second DFT is provided by Δ*q* and the axis scale is developed as:

(A3) $$\Delta q=\frac{\frac{1}{\Delta f}}{\frac{N}{2}}=\frac{2}{N\Delta f}=\frac{2}{f_{s}}$$

(A4) $${\text{axis}}_{2\text{nd}\;\text{DFT}}\left(r\right)=r\Delta q=\frac{2r}{f_{s}};r=\left\{r\in \mathbb{Z}|0\leq r\leq \frac{N}{4}\right\}$$

Note that the length of the second DFT is half of the first transform. According to the preceding equation, the second DFT axis indicates the scaled time and an inverse operation is required to obtain the fundamental frequency as follows:

(A5) $$f_{\text{fund}}\left(r\right)=\frac{1}{\text{axi}s_{2\text{nd}\;\text{DFT}}\left(r\right)}=\frac{f_{s}}{2r};r=\left\{r\in \mathbb{Z}|0<r\leq \frac{N}{4}\right\}$$

## Appendix B.

### Configuration of the Acoustic Experiments

The spectral exploration is executed and analyzed in an anechoic chamber that has been verified to exhibit partial conformance with ISO 3745 for free field and hemi-free field conditions [32]. The speaker (HS80M, Yamaha, Hamamatsu, Japan) generates the designated sound from the computer-connected audio device (Quad-Capture, Roland, Hamamatsu, Japan) that also records sound from the microphone (ECM8000, Behringer, Tortola, British Virgin Island). The computer software for mixing and capturing is a Sonar X2 Studio from Cakewalk (Boston, MA, USA). The overall equipment connection is illustrated in Figure A1a, and the structure is placed between the speaker and microphone. The distance between the microphone end and the speaker driver is 1.2 m. The structure is suspended in front of the microphone with a vertical pole surrounded by an acoustic form; therefore, the structure levitates freely in the acoustically-transparent condition. Figure A1b provides the perspective sketch of the anechoic chamber for overall configuration.

## Appendix C.

### Parameter Description of the Conical Horn Model

This appendix is generated from Donskoy and Cray paper for Equation (7) [30]. Subscripts 1 and 2 indicate throat and mouth position respectively:

(C1) P=ZvS

*P*: sound pressure

*Z*: acoustic impedance

*v*: particle velocity

*S*: surface area

(C2) $$\frac{P_{1}}{P_{0}}=Z_{01}\frac{v_{1}}{v_{0}}S_{1}$$

*P*_0_: sound pressure in the unperturbed medium

*Z*_01_: throat output impedance

*v*_0_: particle velocity in the unperturbed medium

Sound pressure amplification factor:

(C3) $$\begin{array}{c} \frac{P_{1}}{P_{0}}=\rho_{0}c_{0}\;\left(\frac{1-\left(a-cZ_{02}\right)e^{-jkl}}{a+\frac{b}{Z_{01}}-cZ_{02}-d\frac{Z_{02}}{Z_{01}}}\right)\\ a=\frac{x_{1}\sin\left(k\left(l+\varepsilon_{1}\right)\right)}{x_{2}\sin\left(k\varepsilon_{2}\right)}\\ b=-\text{j}\frac{\rho_{0}c_{0}}{S_{1}}\frac{x_{1}}{x_{2}}\sin\left(kl\right)\\ c=-\text{j}\frac{S_{2}}{\rho_{0}c_{0}}\frac{x_{1}}{x_{2}}\frac{\sin\left(k\left(l+\varepsilon_{1}-\varepsilon_{2}\right)\right)}{\sin\left(k\varepsilon_{1}\right)\sin\left(k\varepsilon_{2}\right)}\\ d=-\frac{S_{2}}{S_{1}}\frac{x_{1}}{x_{2}}\frac{\sin\left(k\left(l-\varepsilon_{2}\right)\right)}{\sin\left(k\varepsilon_{2}\right)} \end{array}$$

ρ_0_: air density 0.00120 g/cm^3^ at 20 °C

*c*_0_: sound speed 34,321 cm/s at 20 °C

(C4) $$\text{k}=\frac{2\pi f}{c_{0}}$$

*x*_1_: throat position from vertex

*x*_2_: mouth position from vertex

*l*: conical horn length (*x*_2_ − *x*_1_)

(C5) $$\begin{array}{c} \text{k}\varepsilon_{1}=\tan^{-1}\left(kx_{1}\right)\\ \text{k}\varepsilon_{2}=\tan^{-1}\left(kx_{2}\right) \end{array}$$

*S*_1_: cross section area of throat

*S*_2_: cross section area of mouth

*Z*_01_: throat output impedance

*Z*_02_: mouth output impedance

The radiation impedance is assumed to be equivalent to the impedance of a piston of infinitesimal thickness and zero mass set in the opening of the infinite baffle, given as follows:

(C6) $$\begin{array}{c} Z_{01}=-\frac{\rho_{0}c_{0}}{\pi R_{1}^{2}}\left[1-\frac{J_{1}\left(2kR_{1}\right)}{kR_{1}}+j\frac{H_{1}\left(2kR_{1}\right)}{kR_{1}}\right]\\ Z_{02}=-\frac{\rho_{0}c_{0}}{\pi R_{2}^{2}}\left[1-\frac{J_{1}\left(2kR_{2}\right)}{kR_{2}}+j\frac{H_{1}\left(2kR_{2}\right)}{kR_{2}}\right] \end{array}$$

*R*_1_: equivalent radius of throat $R_{1}=\sqrt{\frac{s_{1}}{\pi }}$

*R*_2_: equivalent radius of mouth $R_{1}=\sqrt{\frac{s_{2}}{\pi }}$

*J*_1_: first order Bessel function

*H*_1_: first order Struve function

## Appendix D.

### Modelling of the Fundamental Frequency and Radial Length by the Least-Square Method

The mathematical model is suggested to be the rational equation without the cross-section factor. Observe that Equation (5) for a cylindrical pipe has the cross section parameter *d*. *c* is the sound speed and the *l* is the propagated radial length of the ML structure. The constant coefficients *m* and *n* should be found by the following method:

(D1) $$\begin{array}{c} f_{\text{fund}}=\frac{mc}{2\left(l+n\right)}\\ 2l+2n=\frac{mc}{f_{\text{fund}}}\\ \frac{2l}{m}+\frac{2n}{m}=\frac{c}{f_{\text{fund}}} \end{array}$$

This is set into matrix form by collecting the data for each ML direction as follows. Since the ML structure contains 11 pyramidal horns, the *q* value is 11:

(D2) $$\begin{array}{c} \left(\begin{array}{cc} l_{1} & 1\\ l_{2} & 1\\ \vdots & \vdots \\ l_{q} & 1 \end{array}\right)\left(\begin{array}{c} 2/m\\ 2n/2m \end{array}\right)=c\left(\begin{array}{c} 1/f_{{\text{fund}}_{1}}\\ 1/f_{\text{fund}2}\\ \vdots \\ 1/f_{{\text{fund}}_{q}} \end{array}\right)\\ \text{Let}\;R=\left(\begin{array}{cc} l_{1} & 1\\ l_{2} & 1\\ \vdots & \vdots \\ l_{q} & 1 \end{array}\right);\;c=\left(\begin{array}{c} 2/m\\ 2n/2m \end{array}\right);\;F=\left(\begin{array}{c} 1/f_{{\text{fund}}_{1}}\\ 1/f_{\text{fund}2}\\ \vdots \\ 1/f_{{\text{fund}}_{q}} \end{array}\right)\\ RC=cF\\ C=c{\left(R^{T}R\right)}^{-1}R^{T}F\\ m=\frac{2}{C\left(1\right)}n=\frac{C\left(2\right)}{C\left(1\right)} \end{array}$$

where *C*(1) and *C*(2) are the first and second element of the *C* matrix, respectively.

## References

[1] Blauert J. (1996). Spatial Hearing: The Psychophysics of Human Sound Localization.

[2] Batteau D.W. (1967). The role of the pinna in human localization. Proc. R. Soc. Lond. Ser. B. Biol. Sci..

[3] Shaw E.A.G., Teranishi R. (1968). Sound pressure generated in an external-ear replica and real human ears by a nearby point source. J. Acoust. Soc. Am..

[4] Gardner M.B., Gardner R.S. (1973). Problem of localization in the median plane: Effect of pinnae cavity occlusion. J. Acoust. Soc. Am..

[5] Hebrank J., Wright D. (1974). Spectral cues used in the localization of sound sources on the median plane. J. Acoust. Soc. Am..

[6] Wright D., Hebrank J.H., Wilson B. (1974). Pinna reflections as cues for localization. J. Acoust. Soc. Am..

[7] Searle C.L., Braida L.D., Cuddy D.R., Davis M.F. (1975). Binaural pinna disparity: Another auditory localization cue. J. Acoust. Soc. Am..

[8] Musicant A.D., Butler R.A. (1984). The influence of pinnae-based spectral cues on sound localization. J. Acoust. Soc. Am..

[9] Asano F., Suzuki Y., Sone T. (1990). Role of spectral cues in median plane localization. J. Acoust. Soc. Am..

[10] Chen J., Van Veen B.D., Hecox K.E. (1992). External ear transfer function modeling: A beamforming approach. J. Acoust. Soc. Am..

[11] Wightman F.L., Kistler D.J. (1997). Monaural sound localization revisited. J. Acoust. Soc. Am..

[12] Iida K., Itoh M., Itagaki A., Morimoto M. (2007). Median plane localization using a parametric model of the head-related transfer function based on spectral cues. Appl. Acoust..

[13] Harris J.G., Pu C.-J., Principe J.C. (2000). A monaural cue sound localizer. Anal. Integr. Circuits Signal Process..

[14] Kumon M., Shimoda T., Kohzawa R., Mizumoto I., Iwai Z. Audio Servo for Robotic Systems With Pinnae.

[15] Nakashima H., Mukai T. 3d Sound Source Localization System Based on Learning of Binaural Hearing.

[16] Tomoko S., Toru N., Makoto K., Ryuichi K., Ikuro M., Zenta I. Spectral Cues for Robust Sound Localization with Pinnae.

[17] Hwang S., Park Y., Park Y.-S. (2011). Sound direction estimation using an artificial ear for robots. Robot. Auton. Syst..

[18] Lee S., Park Y., Choi J.-S. (2014). Estimation of multiple sound source directions using artificial robot ears. Appl. Acoust..

[19] Schillebeeckx F., de Mey F., Peremans H. Bio-Inspired Sonar Antennae: Enhancing Directivity Patterns for localization.

[20] Takiguchi T., Sumida Y., Takashima R., Ariki Y. (2009). Single-channel talker localization based on discrimination of acoustic transfer functions. Eurasip J. Adv. Signal Process..

[21] Takashima R., Takiguchi T., Ariki Y. Hmm-Based Separation of Acoustic Transfer Function for Single-Channel Sound Source Localization.

[b22-sensors-15-03872] Takashima R., Takiguchi T., Ariki Y. (2013). Dimensional feature weighting utilizing multiple kernel learning for single-channel talker location discrimination using the acoustic transfer function. J. Acoust. Soc. Am..

[b23-sensors-15-03872] Takashima R., Takiguchi T., Ariki Y. (2010). Monaural sound-source-direction estimation using the acoustic transfer function of a parabolic reflection board. J. Acoust. Soc. Am..

[24] Friedland G., Yeo C., Hung H. (2010). Dialocalization: Acoustic speaker diarization and visual localization as joint optimization problem. ACM Trans. Multimed. Comput. Commun. Appl..

[25] Stoica P., Moses R. (2005). Spectral Analysis of Signals.

[26] Bogert B.P., Healy M.J.R., Tukey J.W., Rosenblat M. (1963). The quefrency alanysis of time series for echoes: Cepstrum, pseudo-autocovariance, cross-cepstrum, and saphe-cracking. Proc. Symp. Time Series Analysis.

[27] Kim K., Choi A. (2012). Binaural sound localizer for azimuthal movement detection based on diffraction. Sensors.

[28] Kim K. (2013). Lightweight filter architecture for energy efficient mobile vehicle localization based on a distributed acoustic sensor network. Sensors.

[29] Kinsler L.E., Frey A.R., Coppens A.B., Sanders J.V. (2009). Fundamentals of Acoustics.

[b30-sensors-15-03872] Donskoy D.M., Cray B.A. (2012). Acoustic particle velocity horns. J. Acoust. Soc. Am..

[31] Olson H.F. (1957). Acoustical Engineering.

[32] Kim K. (2012). Design and analysis of experimental anechoic chamber for localization. J. Acoust. Soc. Korea.

